# Extended Averaged Learning Subspace Method for Hyperspectral Data Classification

**DOI:** 10.3390/s90604247

**Published:** 2009-06-03

**Authors:** Hasi Bagan, Wataru Takeuchi, Yoshiki Yamagata, Xiaohui Wang, Yoshifumi Yasuoka

**Affiliations:** 1 Center for Global Environmental Research, National Institute for Environmental Studies, 16-2 Onogawa, Tsukuba-City, Ibaraki, 305-8506, Japan; E-mails: hasi.bagan@nies.go.jp (H.B); yamagata@nies.go.jp (Y.Y.); yyasuoka@nies.go.jp (Y.Y.); 2 Institute of Industrial Science, University of Tokyo, Meguro-ku, Tokyo, 153-8505, Japan; E-mail: wataru@iis.u-tokyo.ac.jp (W.T.); 3 Department of Mathematics, University of Texas-Pan American, Edinburg, Texas 78539, USA; E-mail: xhwang@utpa.edu (X.W.)

**Keywords:** hyperspectral, remote sensing, subspace method, averaged learning subspace method, dimension reduction, land cover, classification, normalization

## Abstract

Averaged learning subspace methods (ALSM) have the advantage of being easily implemented and appear to outperform in classification problems of hyperspectral images. However, there remain some open and challenging problems, which if addressed, could further improve their performance in terms of classification accuracy. We carried out experiments mainly by using two kinds of improved subspace methods (namely, dynamic and fixed subspace methods), in conjunction with the [0,1] and [-1,+1] normalization methods. We used different performance indicators to support our experimental studies: classification accuracy, computation time, and the stability of the parameter settings. Results are presented for the AVIRIS Indian Pines data set. Experimental analysis showed that the fixed subspace method combined with the [0,1] normalization method yielded higher classification accuracy than other subspace methods. Moreover, ALSMs are easily applied: only two parameters need to be set, and they can be applied directly to hyperspectral data. In addition, they can completely identify training samples in a finite number of iterations.

## 1. Introduction

Hyperspectral data provide detailed spectral information about ground scenes based on a huge number of channels with narrow contiguous spectral bands. Hyperspectral data can therefore better discriminate the spectral signatures of land-cover classes that appear similar when viewed by traditional multispectral sensors [1]. If successfully exploited, hyperspectral data can yield higher classification accuracy and more detailed class taxonomies.

However, this increase of data dimensionality has introduced challenging methodological problems because of the incapacity of common image processing algorithms to deal with such high-volume data sets [2,3]. In the context of supervised classification, the most common problem is the Hughes phenomenon [4], implies that the required number of training samples for supervised classification increases as a function of dimensionality. One possible solution for mitigating the effects of the Hughes phenomenon is to reduce the dimensionality of the data but at the same time keep as much information as possible. For example, commonly used dimensionality reduction methods include feature selection and feature extraction methods [5-9], principal components analysis (PCA) with conventional classification methods [10], Minimum Noise Fraction [11], orthogonal subspace projection classification methods [12], support vector machine (SVM) classifiers [13-18], and spectral angle mapper and spectral information divergence methods [19,20].

The subspace pattern recognition method is another dimensionality reduction method that can achieve dimension reduction and classification concurrently. The subspace method represents each class by a model of a linear subspace of a feature space. This method was originally proposed by Watanabe *et al.* [21]. In the subspace method, the original high-dimensional data are projected onto a low-dimensional space as done in PCA, but different classes are forced to follow different directions in this low-dimensional space. Subspace analysis has attracted much attention in the area of object recognition and character recognition during the last decade, and some examples are shown by Sakano *et al.* [22], and Omachi and Omachi [23].

For character or face image recognition, the processing object is a binary image or a single-band gray-scale image, but for hyperspectral data, the object is a high-dimensional gray-scale image (dimensions equal to the number of bands). Thus, the subspace method must be extended accordingly to hyperspectral data classifications.

In the specific context of hyperspectral data classification, averaged learning subspace methods (ALSM) for hyperspectral data classification have been described by our previous work [24]. The low-dimensional subspaces that can better characterize class information and can precisely distinguish it from other classes simultaneously. However, they provide results only by using a method of fixed subspace dimension and do not describe the behavior of the dimension selection or the parameter settings.

Moreover, several critical issues are still unclear, for example, (1) how the data normalization method affects the subspace method, (2) how various approaches for selecting subspace dimensions affect the classification accuracy, (3) how learning parameters influence the training speed and classification accuracy, (4) how the size of the training data set influences the classification accuracy, and (5) how to compute eigenvalues from the correlation matrices.

To avoid overflow problems, high-dimensional hyperspectral data need to be normalized to unit-length before one performs the subspace training and classification procedure. The primary objective of image normalization is to remove the effects of outliers by limiting the extent of scatterplot data [25]. Some methods have been proposed for the normalization of satellite data for this purpose [26]. We modified two commonly used normalization methods for hyperspectral data. A detailed description of the normalization methods will be addressed later.

Another major problem with subspace methods regards eigenvalue computation algorithms. The computational cost of subspace methods critically depends on the eigenvalue computation methods; thus, we adopted the QR method [27] instead of the Jacobi method [28].

In this paper, we present the dynamic subspace dimension method, which sets each subspace dimension independently in ALSMs (hereafter referred to as the dynamic subspace method), and the fixed subspace dimension method, which fixes subspace dimensions for each class as the same value as that used in ALSMs (hereafter referred to as the fixed subspace method) based on two normalization methods. We also carried out experimental studies on 16 land-cover classes using the “Indian Pines” 92AV3C9 data set collected from the Airborne Visible/Infrared Imaging Spectrometer (AVIRIS) hyperspectral sensor of June 1992 for the Indian Pines area, Indiana, USA (http://dynamo.ecn.purdue.edu/~biehl/MultiSpec) [29]. Different performance indicators are used to support our experimental analysis, namely, the classification accuracy, computational cost, stability of dimensions selection, and learning parameter settings. Experimental results confirm the considerable advantage of the subspace method in the context of hyperspectral data classification. Since many previously published classification methods used the Indian Pines data sets for experiments, e.g., SVM methods [5,13,14] and feature selection and feature extraction methods [6,8,17]. Therefore it is convenient for the reader to compare the proposed subspace methods described herein with those approaches

The rest of this paper is organized as follows. First, we describe the main idea of subspace methods. Next, we present the data sets and associated processing steps, i.e., normalization methods and eigenvalue computation algorithms. Then, we show comparison results and analyses for AVIRIS hyperspectral data experiments between different normalization methods and our subspace methods. Finally, we present concluding remarks.

## 2. Subspace Methods

### 2.1. CLAFIC and ALSM Subspace Methods

Subspace methods have been extended in many ways. The most basic is called class-featuring information compression (CLAFIC) [30], the procedure of which is as follows.

Assume that available hyperspectral data from a given site contain *n* bands, the implicit pixels are of an *n*-dimension column vector, and user-defined classes of *ω*^(1)^, *ω*^(2)^, …, *ω*^(*K*)^ appear. A set of labeled pixels for all such classes should also be available, divided into training and test data sets.

Given a set of training samples *s_k,i_*∈*R^n^* (1 ≤ *i* ≤ *p*) that belong to class *ω*^(*k*)^ (1 ≤ *k* ≤ *K*), where *n* same as the number of bands in a hyperspectral data set, *p* represents the total number of training samples in class *ω*^(*k*)^, and *K* denotes the number of classes, let *r_k_* denote the number of dimension of the subspace *D^k^*(⊂ *R^n^*) of class *ω*^(*k*)^ for which *r_k_* < min(*n*, *p*) is satisfied. Let *T_k_*=(*t*_*k*,1_, …, *t_k,k_*) denote the base vectors matrix of subspace, where *t_k,i_* is the *i*-th normal orthogonal base.

*D^k^* is included in the subspace spanned by the training sample *s_k,i_* (1 ≤ *i* ≤ *p*), thus the bases can be represented by

(1) $$t_{k,i}=\sum_{j=1}^{p}u_{k,i,j}s_{k,j}=S_{k}U_{k,i}$$

(2) $$T_{k}=\left(t_{k,1},\cdots ,t_{k,k}\right)=(S_{k}U_{k,1},\cdots ,S_{k}U_{k,k})=S_{k}U_{k}$$

Where:

(3) $$S_{k}=(s_{k,1},\cdots ,s_{k,p})$$

(4) $$U_{k,i}={\left(u_{k,i,1},\cdots ,u_{k,i,p}\right)}^{T}$$

(5) $$U_{k}=(U_{k,1},\cdots ,U_{k,k})$$

in which *u_k,i,j_* are coefficient parameters and *U_k,i_*=(*u*_*k,i*,1_, …, *u_k,i,p_*)*^T^* are coefficient vectors.

For the recognition (classification) task, one needs to compute the distance between the pattern vector (pixel) *v* and each subspace, and label *v* into the classes that have the shortest distances. It is formulated by:

(6) $$d(v,\omega^{k})=\underset{1\leq k\leq K}{\arg\min}{\left\|v-T_{k}{\text{T}}_{k}^{T}v\right\|}^{2}=\underset{1\leq k\leq K}{\arg\min}({\left\|v\right\|}^{2}-{\left\|{\text{T}}_{k}^{T}v\right\|}^{2})$$

which becomes:

(7) $$f_{k}(v)=\underset{1\leq k\leq K}{\arg\max}{\left\|{\text{T}}_{k}^{T}v\right\|}^{2}$$

Hence, finding the shortest distance is equivalent to finding the largest squared length of the orthogonal projection between pattern vector *v* and each subspace.

Combining equations (2) and (7), we get:

(8) $$f_{k}(v)=\underset{1\leq k\leq K}{\arg\max}{\left\|{\text{U}}_{k}^{T}\;{\text{S}}_{k}^{T}v\right\|}^{2}$$

where *S_k_^T^v* is a dot product matrix between the pattern vector *v* and the training sample matrix. Equation 8 is equivalent to finding *U_k_* that maximizes:

(9) $$\sum_{i=1}^{p}{\left\|{\text{U}}_{k}^{T}\;{\text{S}}_{k}^{T}s_{k,i}\right\|}^{2}$$

subject to:

(10) $${\text{U}}_{k}^{T}\;{\text{S}}_{k}^{T}\;S_{k}\;U_{k}=I$$

where *I* is an n×n unit matrix. The optimal solution to this problem is given by the following theorem.

#### Theorem

Let *P_k_* = *S_k_^T^S_k_* and let the first *r_k_* largest corresponding eigenvalues be arranged in descending order: *λ*_*k*, 1_≥*λ*_*k*, 2_≥, …, ≥*λ_k_, r_k_* (>0). Let the corresponding eigenvectors be denoted by *α*_*k*,1_, *α*_*k*,2_, *α_k_, r_k_*. The optimum solution of equation (9) is:

(11) $$U_{k}=A_{k}{\left(L_{k}\right)}^{-1}$$

Where:

(12) $$A_{k}=(a_{k,1},a_{k,2},\cdots ,a_{k,r_{k}})$$

(13) $$L_{k}=\text{diag}\;(\sqrt{\lambda_{k,1}},\sqrt{\lambda_{k,2}},\cdots ,\sqrt{\lambda_{k,r_{k}}})$$

The proof is shown by Tsuda [31].

In summary, determining the subspace of class *ω*^(*k*)^ is to solve the eigenvalue problem of matrix *S_k_^T^S_k_*. Here *S_k_^T^S_k_* is the sample correlation matrix from those whose eigenvalues and eigenvectors can be computed by some existing method such as the Jacobi or QR method. The eigenvectors *α*_*k*,1_, *α*_*k*,2_, *α_k_, r_k_* of *S_k_^T^S_k_* are computed corresponding to the first *r_k_* largest eigenvalues. Then these eigenvectors comprise the subspace *D^k^* of *k*-th class *ω*^(*k*)^.

CLAFIC has the drawback that subspaces obtained for one class are not dependent on subspaces of other classes. To avoid this problem, an iteration-learning algorithm, called the ALSM has been proposed [30, 32]. In this method, the subspaces are suitably rotated in each iteration training step. When an error occurs in the ALSM, the correct subspace is rotated toward the misclassified vector and the wrong subspace is rotated away from it. This is achieved by modifying the class conditional correlation matrices and then updating the basis vectors of subspaces.

At each step *k*, one divides the misclassified training samples into two types: either a sample vector of class *ω*^(*i*)^ is misclassified into another class, say *ω*^(*j*)^, or a sample vector of another class, say *ω*^(*k*)^, is misclassified into class *ω*^(*i*)^. We denote the conditional correlation matrix by:

(14) $$P_{k}^{\left(i,j\right)}=\sum_{s_{i,l}}\left\{s_{i,l}s_{i,l}^{T}|s_{i,l}\in \omega^{\left(i\right)},s_{i,l}\mapsto \omega^{\left(j\right)}\right\}$$

where the symbol ↦ denotes the sample that has been misclassified into class *ω*^(*j*)^. Based on current existing subspaces, all training samples are classified according to equation (7), and all matrices *P_k_*^(*i,j*)^, *i, j* = 1, 2, …, *K* are computed. Then, the correlation matrices for each class are computed as:

(15) $$P_{k}^{\left(i\right)}=P_{k-1}^{\left(i\right)}+\alpha \sum_{j\neq i}P_{k}^{\left(i,j\right)}-\beta \sum_{j\neq i}P_{k}^{\left(j,i\right)}$$

where *α* and *β* are the learning parameters, which are usually set to two constant values and do not vary in the iteration process. Then a new subspace of class *ω*^(*i*)^ can be computed from *P_k_*^(^*^i^*^)^.

### 2.2. Subspace Dimension

The subspace dimension markedly affects the pattern recognition rate. The dimensionalities of class subspaces are decided in the CLAFIC stage and then are kept constant during the learning process. Methods for selecting the dimension can be divided into two types: (1) fixed subspace methods, which set a uniform dimension for all classes, and (2) dynamic subspace methods, which set subspace dimensions differently for each class.

For dynamic subspace methods, the selection of the dimension *r_i_* (1 ≤ *I* ≤*c*) of each subspace *ω*^(*i*)^ can be chosen based on a fidelity value (i.e., threshold) *η* (0 < *η* ≤ 1) as follows:

(16) $$\left(\sum_{j=1}^{r_{i}}\lambda_{i,j})/(\sum_{j=1}^{n}\lambda_{i,j})\leq \eta \leq (\sum_{j=1}^{r_{i}+1}\lambda_{i,j})/(\sum_{j=1}^{n}\lambda_{i,j}\right)$$

where eigenvalues are sorted in descending order. The fidelity value decides the degree of overlap between the subspaces. The classification accuracy is sensitive against the fidelity value.

## 3. Preprocessing Methods and Data Sets

In this section, we compare the proposed ALSM classification systems with two different normalization methods developed for ALSM. In the two normalization methods, we use them to normalize each pixel to a unit-length vector by dividing each element according to the vector length. This method can avoid the influence of noise pixels, since it does not use the values of neighboring pixels. Detailed descriptions of the two normalization methods are as follows.

### 3.1. Normalization Methods

Since high-dimensional hyperspectral data are usually at least 10 bits in size, the cumulative values of original high-dimensional hyperspectral data may cause overflow problems when we compute eigenvalues and eigenvectors from the correlation matrix in the ALSM training process without normalization. Hence, normalization is an important step of the algorithm.

There are many normalization methods. In this section, we only consider the [-1, +1] and [0, 1] normalization methods. The choice of scaling each attribute to the range [-1, +1] or [0, 1] is motivated by the successful application of the method to SVM classifiers [33].

In the [0, 1] normalization method, data are normalized to the range [0, 1] as follows: Given a pixel *s* = (*s*_1_, *s*_2_, …, *s_n_*)*^T^*, a normalized pixel is computed as:

(17) $$s^{\prime }={\left(s_{1}/d,s_{2}/d,\cdots ,s_{i}/d\right)}^{T}$$

where d = sqrt(*s*_1_^2^+ *s*_2_^2^+…+ *s_n_*^2^) denotes the pixel length. Obviously, each element value of the normalized pixel is located within the range [0, 1] and the length of the pixel is 1.

In the [-1, +1] normalization method, data are normalized to the range [-1, +1] and the scale can be adjusted such that the mean of the data is equal to zero. The [-1, +1] normalization procedure is given as follows: Given a pixel *s* = (*s*_1_, *s*_2_, …, *s_n_*)*^T^*, we compute:

(18) $$m=\frac{1}{n}\sum_{i=1}^{n}s_{i}$$

(19) $$s_{i}^{\prime }=\left(s_{i}-m\right){\left(\sum_{i=1}^{n}{\left(s_{i}-m\right)}^{2}\right)}^{-0.5}$$

where *s*′ = (*s*′_1_, *s*′_2_, …, *s*′*_n_*)*^T^* denotes the normalized pixel. Note that *s*′_1_+*s*′_2_+…+*s*′*_n_* = 0 if (*s*′_1_-*m*)^2^+(*s*′_2_-*m*)^2^+ …+(*s*′*_n_-m*)^2^ = 0.

### 3.2. Eigenvalue Computation Methods

Computing the eigenvalues and eigenvectors of the correlation matrix of the input data vector (training samples) is a time-consuming process since the correlation matrix can be as large as bands × bands of elements in hyperspectral data. The time can be noticeably shortened by choosing an appropriate eigenvalue computation algorithm.

Let A be an *n*×*n* real or complex matrix whose eigenvalues we seek. The eigenvalue *λ* of *A* satisfies *Ax* = *λx*, and can be computed from the characteristic equation det(*A* − *λI*) = 0. Notice that the correlation matrices in equations (14) and (15) are real symmetric matrices. For a real symmetric matrix, there exists an orthogonal matrix *Q*, such that *Q^T^AQ* = *D*, where *D* is a diagonal matrix. The diagonal elements of *D* are the eigenvalues of *A*, and the columns of *Q* are the corresponding eigenvectors of *A*. The Jacobi and QR methods are two of the most useful algorithms for solving eigenvalue problems. In the Jacobi method, which was originally proposed in 1846, a real symmetric matrix is reduced to a diagonal form by a sequence of plane rotations by orthogonal similarity transformations. The QR method works much faster on a dense symmetric matrix for computing eigenvalues and associated eigenvectors. The basis of the QR method for calculating the eigenvalues of *A* is that an *n* × *n* real symmetric matrix can be written as *A* = *QR* where *Q* is an orthogonal and *R* is an upper triangular matrix. The diagonal elements of *R* are the eigenvalues, and the columns of *Q* are the corresponding eigenvectors. Here, we adopt the *QR* algorithm instead of the Jacobi algorithm for eigenvalue computations. The *QR* algorithm dramatically reduced, by approximately 75%, the time cost of computing eigenvalues in our study. According to recent research, other faster eigenvalue computation algorithms could be adopted [34].

### 3.3. Data Sets and Experimental Settings

To verify the performance of the proposed ALSM algorithm, simulations were carried out on the “Indian Pine” AVIRIS 92AV3C data set, which consists of a 145 × 145 pixel portion [see Figure 7(a)]. The data set was collected over a test site called Indian Pine in northwestern Indiana, USA, by AVIRIS sensors in June 1992. From the 220 original spectral bands, 29 atmospheric water absorption bands (1–3, 103–109, 149–164, and 218–220) were removed, leaving 191 bands. These data values in the scene are proportional to radiance values. Labeled ground truth samples were obtained based on the previous information collected at the Laboratory of Remote Sensing at Purdue University (http://dynamo.ecn.purdue.edu/~biehl/MultiSpec) [see Figure 7(b)] [29]. All 16 land-cover classes available in the accompanying original ground truth were used in our experiments to generate a set of 9,782 pixels for training and testing sets. A simple random sampling method in which each sample had an equal chance of being selected was used for generating training and testing sample sets. Half of the pixels from each class were randomly chosen for training, while the remaining 50% formed the test sets (Table 1).

To avoid the possibility of overflow problems when computing eigenvalues and eigenvectors, all images, training data, and test data were normalized by the [-1,+1] and [0,1] normalization methods. Experiments with various parameter values were necessary to develop a reasonable subspace classifier. The following section describes the design and results these experiments.

## 4. Experimental Results and Discussion

Our objective was to optimize the accuracy of the ALSM classifier by solving the ALSM model selection issue (i.e., estimating the best values for the dimensions and learning parameters). Three types of experiments were carried out to determine how the classification accuracy is affected by the subspace dimension, normalization, and learning parameters. Furthermore, to assess the influence of the number of training data, we further varied the number of training samples drawn from the training set such that 50% of the original training data were used for training while maintaining a constant testing set in the fixed dimension method.

In all experiments, we set a stopping condition of learning iterations as the study data were completely identified. Iterations greater than 1,000 were not considered because learning process failed to converge after 1,000 iterations in our experiments. The proposed ALSM algorithms were developed by using C++ programs (Microsoft Visual Studio 2005.NET).

### 4.1. Subspace Method with [-1, +1] Normalization

#### 1) Influence of the Subspace Dimension

We varied the subspace dimension from small to large. For the fixed subspace method, we varied the dimension from 5 to 9. In the dynamic subspace method, we increased the fidelity value from 0.99985 to 0.99993 in steps of 0.00001. Experimental results indicate that when the fidelity value was smaller than 0.99985, the training process was unable to identify 100% of the training samples within 1000 steps and the classification accuracy dropped. When the fidelity value was greater than 0.99993, the classification accuracy rapidly dropped and caused a divergence. Therefore, we considered only fidelity values within the range [0.99985, 0.99993]. For similar reasons we did not consider dimensions smaller than 5 or greater than 9 in the fixed subspace method. The two learning parameters *α* and *β* in equation (15) were set to the same constant value of 0.3 in both the dynamic subspace and fixed subspace methods.

In the dynamic subspace method, the subspace dimension is determined by equation (16); thus, it varies among different classes. For example, Table 2 lists the subspace dimensions when the fidelity value was set to 0.99986 based on training samples in Table 1. Table 3 shows that the mean of the subspace dimensions increased as the value of the fidelity value was increased.

**Figure 1.**
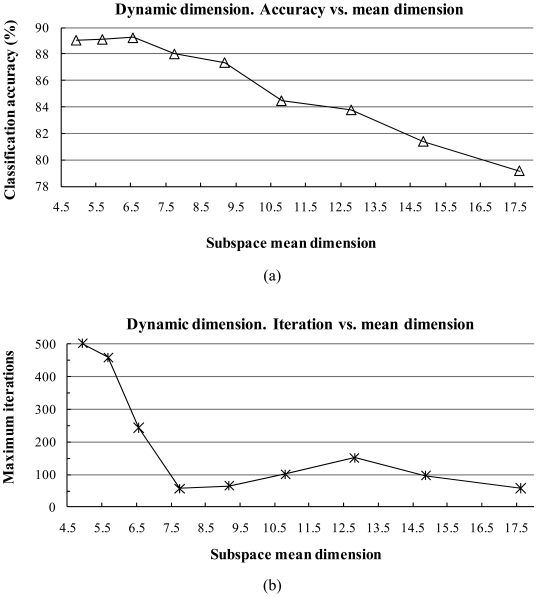

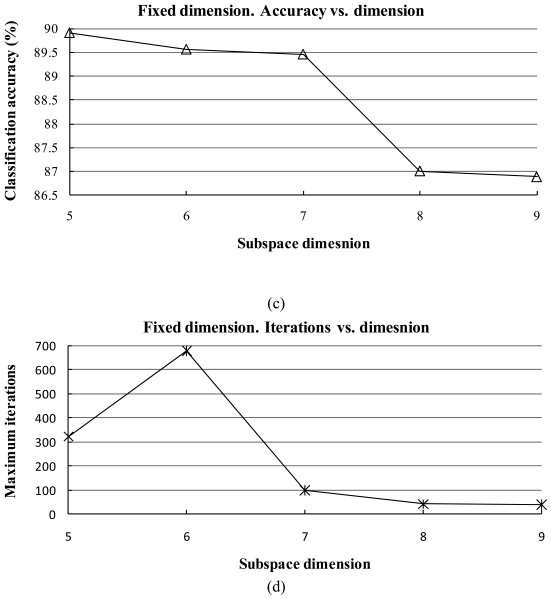
Plots of the classification accuracy and maximum training iterations as a function of the number of subspace dimensions by the dynamic and fixed subspace methods. (a) Classification accuracy by dynamic subspace vs. mean dimension, (b) Maximum learning iterations by dynamic subspace vs. mean dimension, (c) Classification accuracy by fixed subspace vs. subspace dimension, and (d) Maximum learning iterations by fixed subspace vs. subspace dimension.

The classification accuracy and training time (maximum training iterations) as a function of the number of subspace dimensions are shown in Figure 1 for both the dynamic subspace and fixed subspace methods. In the dynamic subspace method [Figures 1(a) and (b)], when the fidelity value was increased, the classification accuracy tended to decrease. However, the study data converged much faster to 100% accuracy than those of small fidelity value; for instance, when the mean dimension value was increased from 4.94 to 17.63, the request time for convergence to 100% accuracy dropped from 502 to 58 iterations. The best classification accuracy in the dynamic subspace method was obtained as 89.25% with a fidelity value of 0.99987 (mean dimension was 6.56).

Similar behavior was also apparent in the fixed subspace method. Figures 1(c) and (d) show that the classification accuracy tended to decrease when the number of dimensions increased. However, there were fewer maximum training iterations when the dimension number increased. The best classification accuracy obtained was 89.91% with the dimension number fixed at 5, which is 0.66% better than the dynamic subspace method.

The classification accuracy decreased when the number of subspace dimensions increased in both the fixed and dynamic subspace methods because of the subspaces overlapping problem. When the fidelity value or subspace dimension increases, subspaces become “closer” or overlap with each other and some noise may be included in the subspace. However, if the fidelity value or the subspace dimension is smaller, subspaces are sufficiently separated, but the smaller the number of subspace dimensions, the more information is required to determine a class.

From the results shown in Figure 1 based on the [-1,+1] normalization method, fixed subspace methods generally yield better classification accuracy than corresponding dynamic subspace methods (89.91% vs. 89.25%).

#### 2) Stability of Learning Parameters

We investigated the sensitivity of the classification accuracy and maximum training iterations to the learning parameters in both the dynamic and fixed subspace methods. The two learning parameters were set to the same constant value; results from using distinct values are discussed later.

**Figure 2.**
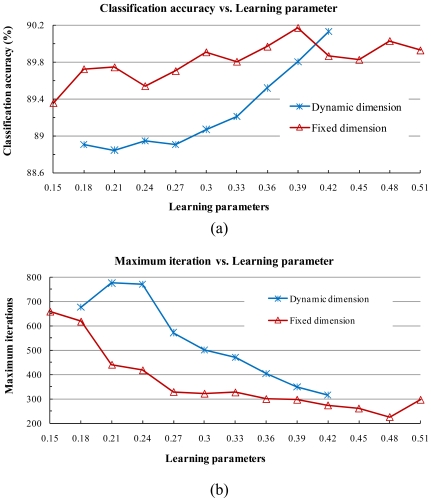
Classification accuracy and maximum training iterations as a function of learning parameters combined with [-1, +1] normalization. (a) Classification accuracy by the dynamic subspace method and classification accuracy by the fixed subspace method (b) maximum learning iterations by the dynamic subspace method and maximum learning iterations by the fixed subspace method. The fidelity value in the dynamic subspace method was 0.99985 and the dimension in the fixed subspace method was 5.

In the dynamic subspace method, the fidelity value was fixed at 0.99985 and the corresponding convergence interval of learning parameters was [0.18, 0.42], implies the two learning parameters were set to the same constant value within [0.18, 0.42]. In the fixed subspace method, the number of dimensions was fixed at 5 and the corresponding convergence interval of learning parameters was [0.15, 0.51]. As shown in Figure 2, the classification accuracy increased stably and the training iterations rapidly lowered when we increased the parameter value in steps of 0.03 in both the dynamic and fixed subspace methods.

#### 3) Influence of Parameters

**Figure 3.**
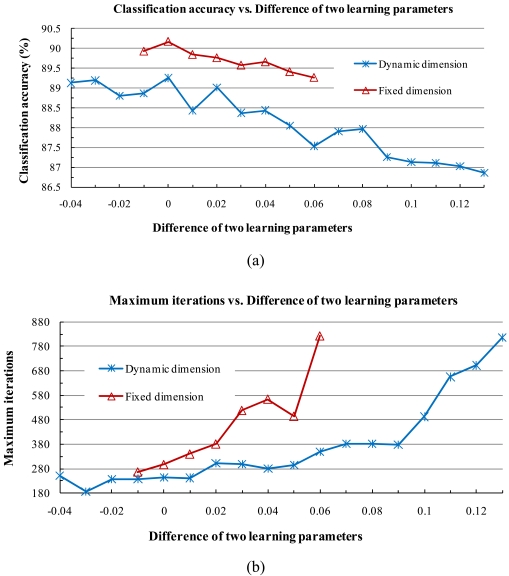
Classification accuracy and maximum iterations vs. the difference between *α* and *β*. **(a)** and **(b)** show the classification accuracy and maximum training iterations, respectively, with various learning parameters by the dynamic subspace method in which *α* was fixed at 0.3 while *β* varied from 0.17 to 0.34; and with various learning parameters by the fixed subspace method in which *α* was fixed at 0.39 while *β* varied from 0.33 to 0.4.

We examined the behavior of the classification accuracy when the two learning parameters were set to different constant values in both the dynamic and fixed subspace methods. In the dynamic subspace method, we set the fidelity value to 0.99987 and the learning parameter *α* to 0.3, and then varied the learning parameter of *β* in steps of 0.01. The corresponding convergence interval of *β* was [0.17, 0.34]. In the fixed subspace method, we set the number of dimensions to 5, set *α* to 0.39, and varied *β* in steps of 0.01, then the corresponding convergence interval of *β* became [0.33, 0.4] (Figure 3).

As shown in Figure 3, when the two learning parameters were set equal or very close to each other, our subspace method converged faster and the classification accuracy was higher. Otherwise, the classification accuracy dropped or it took more time to converge in both the dynamic and fixed subspace methods. Since the other dimension and learning parameters exhibited similar behaviors, to ensure clarity of the plots we do not present the result here.

### 4.2. Subspace Method with [0, 1] Normalization

**Figure 4.**
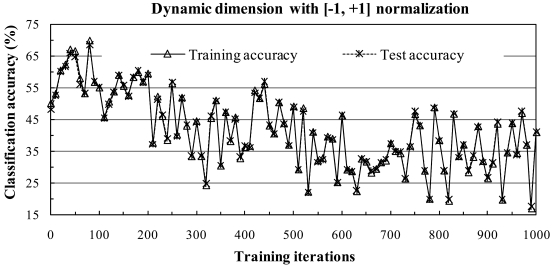
The dynamic subspace method diverged when the [0,1] normalization method was used.

The dynamic subspace method diverged when the [0,1] normalization method was used for various fidelity values and learning parameters. Figure 4 shows an example in which the fidelity value was 0.99986 and learning parameters were set to the single value of 0.3. Thus, hereafter we consider only the fixed subspace method.

#### 1) Behavior of the Two Learning Parameters

**Figure 5.**
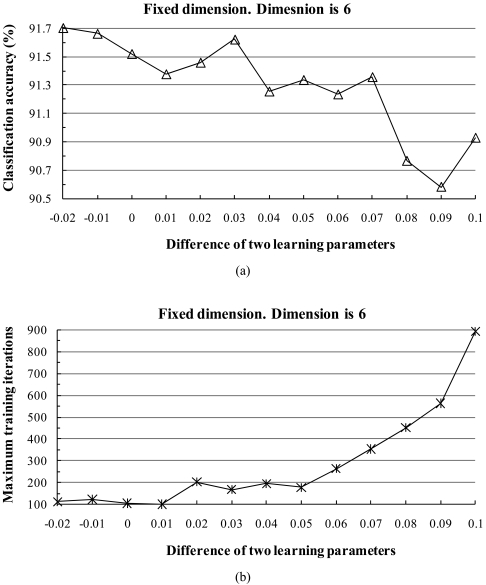
Plots of the difference between *α* and *β* vs. (a) classification accuracy and (b) maximum iterations, provided by the fixed subspace method in conjunction with the [0, 1] normalization method.

First, we examined the behavior of the classification accuracy when the two learning parameters were set to different constant values. Similar to the behavior shown in Figure 3, when the two learning parameters were equal or close to each other, generally the classification accuracy was high and the training time was short. For example, Figure 5 shows the behavior of the classification accuracy and maximum training iterations when the fixed dimension was 6, the parameter *α* was kept to a constant value of 0.45, and the corresponding convergence interval of *β* was [0.35, 0.47]. Similar to Figure 3, we increased the value of the parameter *β* in steps of 0.01 (Figure 5). We found that when the two learning parameters got closer in value or were equal, the classification accuracy increased and our method converged relatively fast and effectively. Otherwise, the classification accuracy decreased or the training samples did not converge.

#### 2) Behavior of Training and Test Sets in Learning Iterations

**Figure 6.**
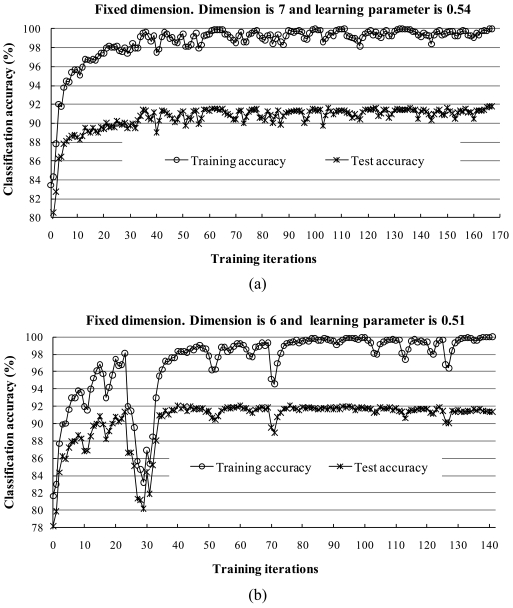
Plots of the accuracy rate vs. the number of iterations for the training and test samples. (a) Dimension of 7 with the learning parameter 0.54; the best test data accuracy of 91.79% was reached when the training iterations completed (at 167). (b) Dimension of 6 with the learning parameter 0.51; an accuracy of 91.34% was achieved when the training iteration completed (at 141). However, the best test data accuracy of 92.11% was reached at training iteration 76.

To assess the training effectiveness at each iteration step, we classified the test samples by concurrently generating subspaces according to equation (7). The test samples were not joined to the training process, but were used only to assess the classification accuracy. Since the other dimensions and learning parameters provided quite similar results, we present only the results of the following two cases: (1) dimension of 7 with both learning parameters set to 0.54, and (2) dimension of 6 with both learning parameters set to 0.51. Figure 6 shows the behaviors of the training and test accuracies by training iteration.

**Figure 7.**
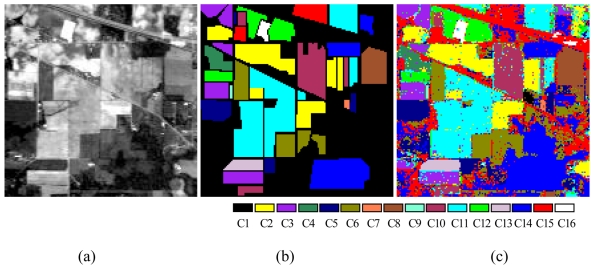
(a) Band 16 (central wavelength: 547.60 nm) of the AVIRIS Indian Pines data set and (b) ground truth. (c) Classification map obtained with the fixed subspace method combined with the [0, 1] normalization method. The subspace dimension was 7 and both learning parameters were 0.54. The overall classification accuracy was 91.79%.

The accuracy of the training and testing data sets increased steadily with the learning iteration (Figure 6). When the training data converged to 100% accuracy, the classification accuracy of the test data steadily increased or was very close to the best. The best test data accuracy of 92.11% was reached at the training iteration 76 for dimension 6 [Figure 6(b)]. Figure 7 shows the classification results with a dimension of 7, and Table 4 presents the corresponding confusion matrix. The matrix scores how the classification process has labeled a series of test sites or test pixels for which the correct land-cover label is known [35,36].

In Table 4, we notice the small training sets of classes 7 and 9, which produced accuracies of 69.23% and 100%, respectively.

#### 3) Behavior of Classification Accuracy and Training Time

**Figure 8.**
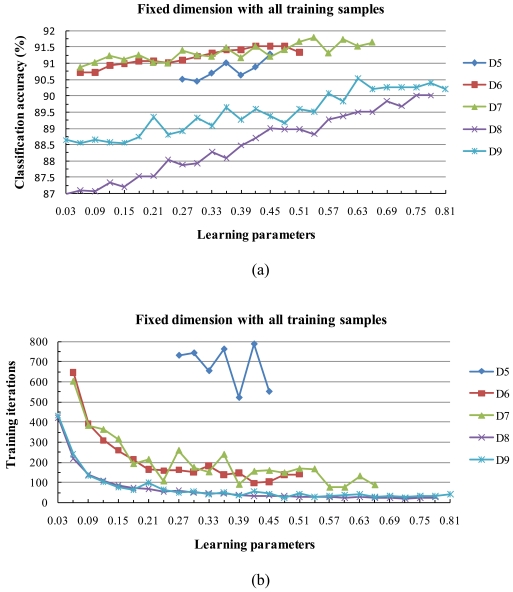
(a) Classification accuracy and (b) maximum training iterations for various subspace dimensions and learning parameters. The highest classification accuracy of 91.79% was reached by D7 with a learning parameter of 0.54; the corresponding maximum training iteration was 167.

Since using two different learning parameters did not improve the classification accuracy, hereafter we do not consider such a case. For subspace dimensions, we consider only those from 5 to 9, since a dimension of less than 5 or more than 9 causes the training process to diverge or the classification accuracy to drop. We increased the learning parameters starting from 0.03 with a step-size of 0.03 in all cases. In Figure 8, the projection of one of the curves in the horizontal axis indicates the convergence interval. For example, In Figure 8(a), the label D7 indicates that the number of dimensions is 7 and the corresponding convergence interval is [0.06, 0.66].

The five curves in Figure 8, show strongly similar stability in their convergence to 100% accuracy in a finite number of steps. However, smaller learning parameter values tended to need more time (iterations) to converge to 100% accuracy. The subspace dimension was not critical to the behavior of the classification accuracy among the values of 5, 6, or 7 [Figure 8(a)], but the smaller dimensions tended to require more training time [Figure 8(b)]. The classification accuracy increased for large values of the learning parameters.

#### 4) Behavior of the Algorithm for Low Sample Sizes

For examining the behavior of the classifiers with respect to the size of the training set, this experiment evaluated the effect of training set size on the performance of fixed subspace method in conjunction with the [0, 1] normalization.

To assess the influence of the number of training data, we reduced the number of training samples by 50% except for classes 7 and 9. For classes 7 and 9, we used the original training data set since the number of training samples was limited. The numbers of training and test samples are listed in Table 5.

**Figure 9.**
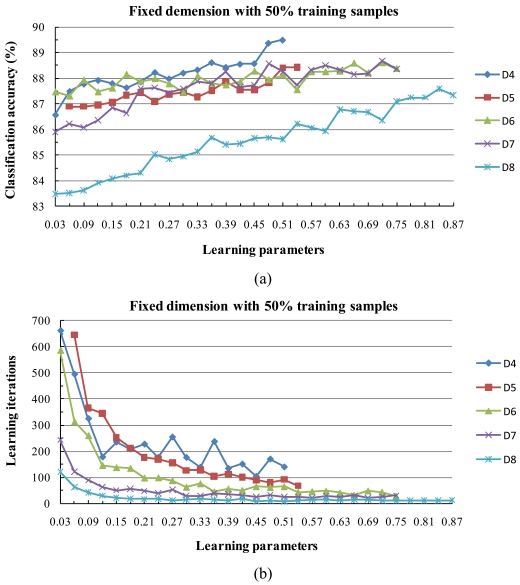
Behavior of the algorithm with low sample sizes. (a) Classification accuracy and (b) maximum training iterations for various subspace dimensions and learning parameters after reducing the number of training samples by 50%. The maximum classification accuracy value was reached at 89.50% in D4 for the learning parameter 0.51; the corresponding maximum training iteration was 141.

Figure 9 shows how the subspace dimension and learning parameters influence classification accuracy and maximum training iterations. The training iterations and learning parameters in Figure 9 are the same as those in the previous study (e.g., Figure 8) but we varied the dimension from 4 to 8 in this case because of the small number of training sets and because the classification accuracy decreases rapidly with dimensions less than 4 or larger than 8.

As expected, the experimental results clearly show the same behavior as in Figure 8. The curves in Figures 8 and 9 show strong behavioral similarity with respect to the stability of convergence to 100% accuracy in a finite number of steps, and when we increased the value of the learning parameters, the training process became convergent more quickly.

Table 6 shows the maximum classification accuracy and the corresponding maximum training iterations from the ALSM classifications for both the full and 50% training data sizes. Classification accuracy results in Table 6 clearly show a positive correlation with the size of the training set. Small training sets can improve the convergent speed but lower the classification accuracy. These results are in agreement with the literature [37,38]. It is notable that one of the advantages of our method is that when the number of subspace dimensions is varied within a small range, the best classification accuracy is not very sensitive to it and the training samples are completely recognized by the generated subspace classifiers. However, most other hyperspectral data classification methods do not describe recognition of the training sample behavior and accuracy.

## 5. Conclusions

In this paper, we have proposed strategies for the optimization of subspace algorithms by modification of the algorithms to make them more suitable for application to hyperspectral data sets. We modified the subspace methods based on the combination of a normalization technique and QR method, and applied them to an AVIRIS dataset to classify 16 land cover classes. Specifically, we verified the following:

1. The fixed subspace method in conjunction with the [0,1] normalization method is substantially more accurate than other approaches such as the dynamic subspace method.
2. When the two learning parameters are equal or close to each other, the classification accuracy increases. When the value of the learning parameters is large, the classification accuracy tends to increase and the training time shortens.
3. The classification accuracy is not sensitive to the dimension of the subspace when it is within at small interval, but a larger dimension tends to reduce the training time.
4. Experimental results clearly showed the classification accuracy increased with the size of the training data set.

Our experiments performed by using the subspace method indicate that it is an effective method: it possesses high-speed convergence and can completely identify training samples. Our findings can provide some guidance for the selection of subspace methods, e.g., effective dimension selection and parameter selection rules that make use of the benefits of subspace classifiers and avoid the weaknesses.

Additional aspects of this method remain to be investigated before it becomes operational. The method needs to be extended by considering a broader spectrum of land-cover classes that might also be aggregated to different informational levels. Moreover, data from different sensors and platforms need to be analyzed to explore the sensitivity and efficiency of our method for handling different spatial and spectral resolutions data. The subspace technique might be further improved by considering several subspaces instead of a single subspace in one class, or by combining with some other innovative methods such as kernel-based methods [39, 40]. Another remaining issue is why the dynamic subspace method does not work well in conjunction with the [-1, +1] normalization method. While beyond the scope of this paper, these issues will direct our future research activities.

## Figures and Tables

**Table 1.** Land-cover classes and number of training and test samples in the AVIRIS indian pines data set.

**Class**	**Training Samples**	**Test Samples**
C1. alfalfa	26	26
C2. corn-notill	671	671
C3. corn-min	400	400
C4. corn	98	99
C5. grass-pasture	228	228
C6. grass-trees	357	357
C7. grass-pasture	13	13
C8. hay-windrowed	241	241
C9. oats	10	10
C10. soybean-notill	480	480
C11. soybean-min	1,137	1,137
C12. soybean-cleantill	282	283
C13. wheat	104	105
C14. woods	617	618
C15. bldg-grass	180	181
C16. stone-steel	44	45
**Total**	**4,888**	**4,894**

**Table 2.** Subspace dimension of each class (see Table 1) with a fidelity value of 0.99986. the subspace mean dimension is 5.69, variance 3.71, and standard deviation 1.93.

C1	C2	C3	C4	C 5	C 6	C7	C8	C9	C10	C11	C12	C13	C14	C15	C16
4	6	7	6	4	7	2	5	2	8	8	8	6	5	8	5

**Table 3.** Mean subspace dimensions for different fidelity values.

Fidelity value	0.99985	0.99986	0.99987	0.99988	0.09989	0.9999	0.99991	0.99992	0.99993
Mean dimension	4.94	5.69	6.56	7.75	9.19	10.81	12.81	14.88	17.63

**Table 4.** Results of the confusion matrix.

**Class**	**1**	**2**	**3**	**4**	**5**	**6**	**7**	**8**	**9**	**10**	**11**	**12**	**13**	**14**	**15**	**16**	**User acc.**
**1**	***23***	0	0	0	0	0	0	2	0	0	0	0	0	0	0	0	92
**2**	0	***618***	10	6	0	0	0	0	0	19	47	2	0	0	0	1	87.91
**3**	0	10	***344***	7	0	0	0	0	0	5	15	11	0	0	0	0	87.76
**4**	0	8	9	***82***	0	0	0	0	0	1	0	1	0	0	0	0	81.19
**5**	0	0	0	0	***224***	0	1	0	0	1	0	0	0	0	1	0	98.68
**6**	0	0	0	0	1	***349***	0	0	0	3	1	1	0	2	2	0	97.21
**7**	0	0	0	0	0	0	***9***	0	0	0	0	0	0	0	0	0	100
**8**	3	0	0	0	0	0	3	***236***	0	0	0	0	0	0	0	0	97.52
**9**	0	0	0	0	0	0	0	0	***10***	0	0	0	0	0	1	0	90.91
**10**	0	7	0	1	0	1	0	0	0	***416***	24	2	0	0	0	1	92.04
**11**	0	27	27	2	2	1	0	0	0	32	***1044***	8	0	0	0	3	91.1
**12**	0	1	10	0	1	0	0	0	0	3	5	***257***	0	0	1	0	92.45
**13**	0	0	0	0	0	0	0	0	0	0	0	0	***105***	0	1	0	99.06
**14**	0	0	0	0	0	1	0	0	0	0	0	0	0	***599***	39	0	93.74
**15**	0	0	0	1	0	5	0	3	0	0	0	1	0	17	***136***	0	83.44
**16**	0	0	0	0	0	0	0	0	0	0	1	0	0	0	0	***40***	97.56
**Total**	26	671	400	99	228	357	13	241	10	480	1137	283	105	618	181	45	
**Prod. acc**	88.46	92.1	86	82.83	98.25	97.76	69.23	97.93	100	86.67	91.82	90.81	100	96.93	75.14	88.89	(%)

Overall classification accuracy: 91.79%; Kappa coefficient: 0.9065

**Table 5.** Numbers of training and test samples from the AVIRIS indian pines data set. Test samples were the same as in Table 1.

Class	C1	C2	C3	C4	C5	C6	C7	C8	C9	C10	C11	C12	C13	C14	C15	C16	Total
Training	13	335	200	49	114	178	13	120	10	240	568	141	52	308	90	22	2453
Test	26	671	400	99	228	357	13	241	10	480	1137	283	105	618	181	45	4894

**Table 6.** Comparisons of the maximum classification accuracy and the corresponding training time with 100% and 50% of training data.

	100% of training data					50% of training data				
Dimension	D5	D6	D7	D8	D9	D4	D5	D6	D7	D8
Classification accuracy	91.28%	91.52%	91.79%	90.01%	90.54%	89.50%	88.43%	88.60%	88.68%	87.60%
Training iterations	553	97	167	33	41	141	68	44	27	11
Learning parameter	0.45	0.42	0.54	0.75	0.63	0.51	0.54	0.72	0.72	0.84
